# Bioactivity-Guided Fractionation of Pine Needle Reveals Catechin as an Anti-hypertension Agent via Inhibiting Angiotensin-Converting Enzyme

**DOI:** 10.1038/s41598-017-07748-x

**Published:** 2017-08-21

**Authors:** Jian He

**Affiliations:** 1 Key Laboratory of Systems Biomedicine (Ministry of Education), Shanghai Center for Systems Biomedicine, Shanghai Jiaotong University, 800 Dongchuan Road, Shanghai, 200240 China; 2 Dalian Institute of Chemical Physics, Chinese Academy of Sciences, Dalian, 116023 China

## Abstract

Hypertension has been recognized as one of the highest risk factors for cardiovascular diseases. Anti-hypertension agent screening and development has been recognized as a pharmaceutical therapy approach for the cardiovascular diseases treatment. Many kinds of traditional Chinese medicines, such as pine needle, have been used for the treatment of hypertension for a long time, but the bioactive ingredients which responsible for their therapeutic effectiveness are remain unclear. Therefore, screening bioactive chemicals in natural sources is still the most straightforward strategy for novel Angiotensin-converting enzyme inhibitor (ACEi)-based anti-hypertension agents discovery. In this study, we demonstrated a bioactivity-guided fractionation strategy for identifying bioactive fractions and chemicals from pine needle based on LC/MS assay as well as elucidating their mechanisms of pharmacological activity. And we found out the compound in pine needle extracts being ACE-inhibitory active is catechin. When ACE activity was assayed in rat tissue membranes, it was observed that catechin demonstrate ACE inhibition in kidney, lung and testes tissue. All these presents catechin in pine needle could be a potential cardiovascular medicine.

## Introduction

**Figure 1:**
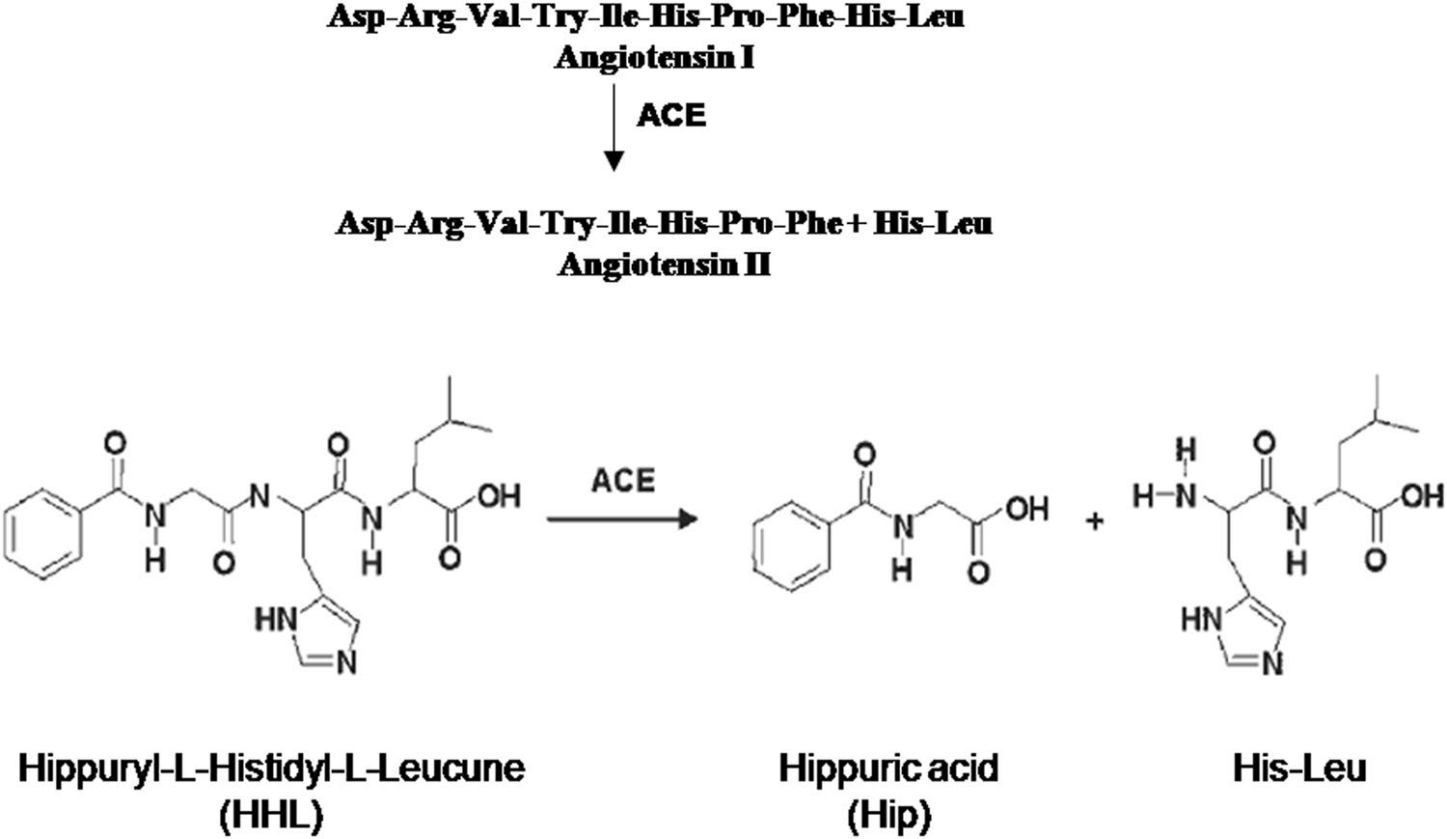
Reactions catalyzed by the Angiotensin-converting enzyme of lung.

Cardiovascular diseases (CVD) are leadings cause of morbidity and mortality in China and around the world, therefore the treatment of CVD has become a public health issue with the most importance^1, 2^. Hypertension has been recognized as one of the highest risk factors for CVD^3–5^. Untreated hypertension could finally develop into CVD, hypertensive retinopathy, stroke, kidney dysfunction, disability and even death^6^. Nowadays there are multiple targets for hypertension treatment; meanwhile there are various types of medications according to the targets including angiotensin receptor blockers, β-adrenoreceptor blockers, angiotensin converting enzyme (ACE) inhibitors, calcium channel blockers, α-adrenoreceptor antagonists, diuretics and centrally acting agents. Among these targets, ACE which subordinating to renin–angiotensin system is a powerful mechanism for controlling blood pressure^7, 8^. The patients with elevated plasma rennin–angiotensin activity demonstrated a five-fold increased incidence of myocardial infarction^3^. ACE (EC 3.4.15.1, dipeptidyl carboxypeptidase) is a glycoprotein peptidyl dipeptide hydrolase which cleaves histidyl-leucine from angiotensin I forming the angiotensin II, which is a potent vasoconstrictor (Fig. 1). Inhibition of ACE has been proven to be an efficient approach to treat the cardiovascular disorders^9^. Therefore, ACE inhibitors (ACEi) have become the preferred agents for the therapies of patients with concurrent secondary diseases^10^. Many synthetic ACEi drugs such as captopril, benazepril and enlapril are currently widely used in hypertension and heart failure treatment, whereas, these classic ACEi exert side effects^11–14^, there will be a huge commercial interest in new, safe chemicals with ACE-inhibiting efficiency. Most recently, many groups have reported the discovery of natural ACEi agents in various natural sources^15–18^, in particular TCMs.

TCMs including many kinds of herb-medicine and dietary therapeutics have been used in clinical treatment for thousands of years. They are widely used in the treatment of various diseases, such as cardiovascular disease, tumor, inflammation^19–21^. The essential of the modernization of TCMs is to confirm the pharmacological activity compounds and their mechanisms of pharmacology^21^. Nowadays, bioactivities from TCMs have become a rich source for medicaments^21–24^, especially secondary metabolites in plants and their derivatives consist of a great part of medicant^20^. Given the numerous biological resources and the rapidly expansion of new technologies such as phenotypic assays and synthetic biology, natural products still remain to be impressivecandidates for drug discovery^24–27^. Nowadays, there has been considerable interest in the potential for using natural product to treat hypertension, especially for people with borderline to mild high blood pressure that does not warrant the prescription of anti-hypertensive drugs^28^. Therefore, screening of natural sources is still a straightforward strategy for the discovery of new ACEi-based anti-hypertension drugs.

In order to study the ACE inhibition, a simple, reliable and rapid method for detecting the inhibitory of ACEi is required. The inhibitory is mainly measured by detecting the conversion ratio of the substrate of ACE in the presence and absence of ACE inhibitors. Instead of Angiotensin I, some artificial substrates are commonly applied for the detection. Most commonly substrate is hippuryl-L-histidyl-L-leucine (HHL) as described by Cheung’s group firstly (Fig. 1)^29^. Scientists modified this method by applying HPLC with UV detection^30, 31^. Van Elswijk’s group invented an alternative approach for the screening of a complex sample applying a HPLC procedure with biochemical detection, which separation and bioactivity detection could be completed in one step^32^. Moreover, these procedures are also suitable for artificial substrates too. The usage of mass spectrometry for measuring enzyme catalyzed reactions has become a good approach to monitor reactions with substrates^33^. However, the quantification by mass spectrometry is still a challenge^34, 35^. In particular for the analysis of complex samples, ESI-MS combined with HPLC is the most straightforward method for the quantitative analysis of small molecules substrate, such as angiotensins^36^.

**Figure 2:**
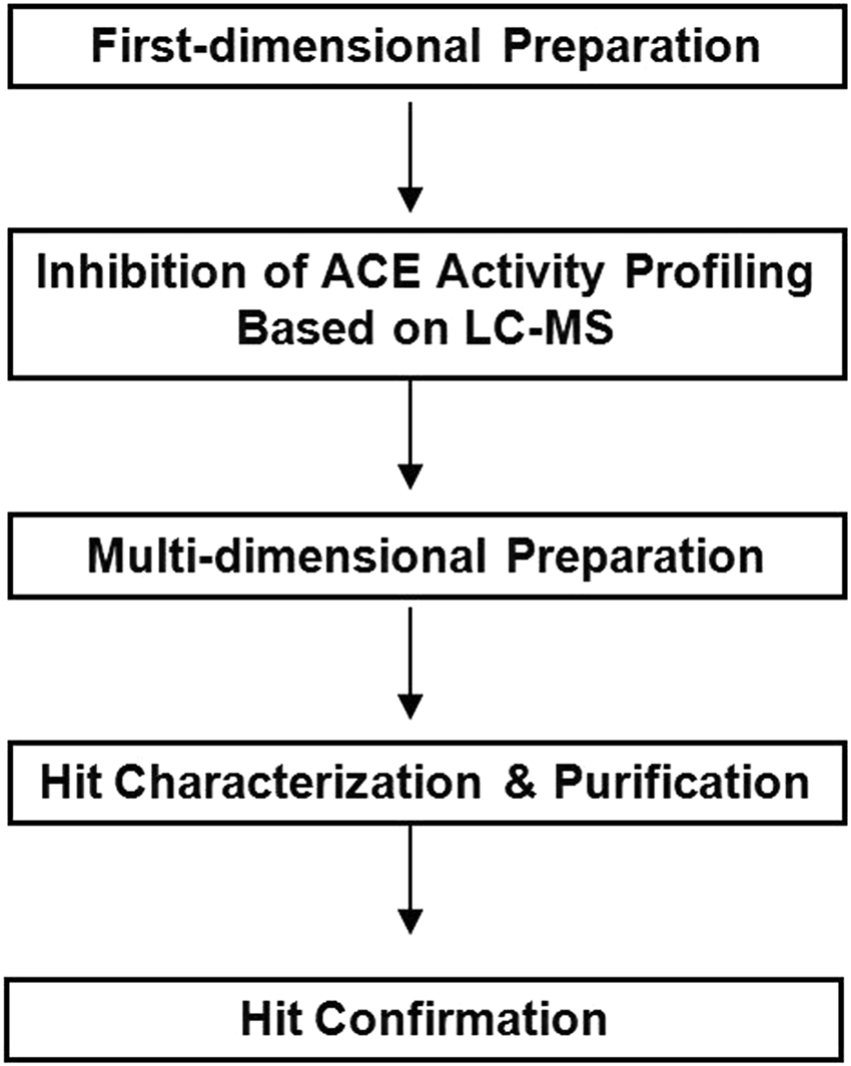
A LC/MS centric strategy for identifying active compounds from pine needle.

In the recent years, bioactivity-guided fractionation has become an attractive approach for drug profiling and screening^37, 38^. In this study, we describe, for the first time, a bioactivity-guided fractionation via ACE Inhibiting profiling based on LC/MS -centric strategy (Fig. 2) to identify bioactive compounds from a TCM, pine needle (PN), and elucidate its bioactive mechanism. Pine has been extensively used in current clinical practice and its bark and oil have been demonstrated good anti-hypertension, antioxidant properties, anti-proliferative effect^39–42^, but the mechanism is still undefined. Pine needle, the leaves of the pine, is a TCM which has showed superior cardiovascular protective effects. But the cardiovascular protective bioactive compounds and mechanisms are also not well understood. Given the complexity of PN fraction and poor sensitivity of conventional spectrum measurement techniques^29, 43^, here we described a modified ACE active assay which is based on LC-MS and elucidated the cardioprotective factor in pine needle is a kind of flavone, which acts by inhibiting the activity of ACE. The presence of this makes pine needle a potential cardiovascular medicine.

## Material and Methods

This study was carried out in accordance with the recommendations of ‘the Guide for the Care and Use of Laboratory Animals of Shanghai Jiao Tong University’. The protocol was approved by the ‘Ethic Committee of Shanghai Jiao Tong University’.

### Materials

ACE from rabbit lung, HHL (Hippuryl-His-Leu), Captopril, Hippuric acid and other reagents were purchased from Sigma. Acetonitrile was HPLC grade.

### Working solution preparation

ACE was diluted in sodium-borate (SSB) buffer (6.18 g boric acid, 17.55 g NaCl in 1 L MiliQ water, pH 8.3) to 500 mU/mL. Aliquots of 100 uL were stored at −20 °C until use. 0.0179 g hippuric acid (Hip) was dissolve in 100 mL MiliQ water, 0.0215 g HHL was dissolve in 10 mL SSB buffer. Captopril was also dissolved in SSB buffer, 1 M HCl was employed as stop solution. All reagents were stored at −20 °C before use.

### Standard curve and linear range assessment

Hip was solved in MiliQ water as the standard solution, the high concentration group are solution with concentrations 5, 2.5, 1.25, 0.625 M, the moderate concentration group are 5, 2.5, 1.25, 0.625 mM and the low concentration group are 5, 2.5, 1.25, 0.625 µM. 15 µL Hip solution of each concentration was performed on a C18 HC column (150 × 3.0 mm i.d., particle size 5 µm), and analytes were detected at λ = 228 nm. The column was eluted with a two solvents system: (A) 0.1% trifluoroacetic acid (TFA) in water and (B) acetonitrile (ACN). The separation linear gradient (time (concentration v%, phase B)) was 0 min (85%, B)−15 min (60%, B)−20 min (10%, B) with a flow rate of 1 ml/min. Each concentration was repeated three times, and then made the standard curve and regression equation based on injection volume X (ug) as the horizontal coordinate and average peak area (Y) from three measurements as the ordinate.

### Accuracy test and recovery assay

Five microlitre 1.0 mg/mL Hip standard solution was injected in the above system five times continually and another five times at five different time points separately in order to be validated for accuracy. 0.1, 1.0, 10.0 ug/mL Hip was injected in the above system five times to measure the recovery rate by measuring the peak area and calculate the relative standard deviation.

### Determination of ACE activity

First, we tested the ACE activity based on the above system. The assay was performed by mixing 5 uL 500mU ACE with 45 uL inhibitor solution or SSB buffer for control. After incubated at 37 °C for 30 min, 12.5 uL of 5 mM HHL solution were added and the sample was further incubated at 37 °C for 10 min with continuous agitation at 450 rpm. The reaction was stopped by addition of 75 uL of 1 M HCl and the solution was filtered through a 0.45-um nylon syringe filter before being analyzed by reversed-phase HPLC.

The HPLC analysis was performed on a C18 HC column (150 × 3.0 mm i.d.), particle size 5 um with a Varian chromatographic system and analytes were detected at the wavelength of λ = 228 nm. The column was eluted at a flow rate of 1 mL/min with a two solvents system: (A) 0.1% TFA in water and (B) acetonitrile (ACN). The separation linear gradient was 0 min (85%, B) – 15 min (60%, B) – 20 min (10%, B) with1 mL/min flow rate.

### ACE inhibition measurement and data visualization and clustering

The calculation of ACE inhibition rate was based on the comparison between the concentration of the product, Hip, with or withnot (control sample) an inhibitor. After process in LC-MS of the control or the inhibitor, the Hip peak areas (S) obtained in the two cases were measured and the percentage of ACE inhibition (I %) was calculated in accordance withthe equation below:

$${\rm{ACE}}\,{\rm{Inhibitionrate}}\,({\rm{I}})=[1-({{\rm{S}}}_{{\rm{inhibitor}}}-{{\rm{S}}}_{{\rm{blank}}})/({{\rm{S}}}_{{\rm{control}}}-{{\rm{S}}}_{{\rm{blank}}})]\times 100 \% ;$$

The inhibition results were extracted for similarity analysis. The responses were visualized to demonstrate the differences in inhibition rate (red: high inhibition rate; black: low inhibition rate). In the fraction-inhibition rate matrix each column represents an inhibition rate of the specific fraction, and each row represents one fraction with three repeats. Each column and row carries equal weight. SSB buffer at an equal concentration to all fractions was employed as a negative control.

### Determination of ACE-inhibitory activity of Captopril

Captopril was used as a reference of ACE inhibitor. The degree of ACE inhibition was measured using different volume of Captopril from 5 to 100 uL of a 5 ng/mL solution. The IC_50_ was determined from the ACE inhibition curve. Three separates experiments were realized to evaluate the IC_50_ standard deviation.

### Extraction, First-dimensional fractionation of pine needle and Analysis

The key fFractionations were performed by using mass-directed purification system (Waters, MA, USA) with a C18ME-20 mm × 250 mm, 5 um column. We loaded 0.5 mL of each fractionation on this system. The mobile phase used was 0.1% (v/v) formic acid (FA) in ACN (phase A) and 0.1% (v/v) FA in water (v/v) (phase B). The separation linear gradient was 0 min (95%, B) −30 min (70%, B) – 45 min (10%, B). The flow rate was 20 mL/min. Total ion and UV at 280 nm were used for detection. We collected the fractions from 2 min till 45 min, and each fraction was collected every two minutes, the last two fractions were combined together. Total 20 fractions were collected for each extraction. Before the fractions were aliquot and dried in a vacuum condition, they were analyzed in the same system above with flow rate 1 ml/min. The dried fractions were then dissolved in water to 2 mg/ml, and diluted in SSB buffer before profiling.

### Determination of ACE-inhibitory activity of PN first-dimensional fractionations

For Captopril, IC_50_ values were determined from five ACE inhibition curves obtained with different amounts of a same batch of PN fractionations or secondary fractionations of the active fraction. Other batches of the PN fractionations were then measured in regard to ACE inhibition to measure the reproducibility of the process.

### Secondary fractionation of the active fractions

After identification, the bioactive fraction was subject to secondary fractionation by using purification system same as in the first fractionation procedure. The active fraction was subjected to secondary fractions by using the C18 ME column (Acchrom, China). The mobile phase was 0.1% (v/v) FA in ACN (phase A) and 0.1% (v/v) FA in water (v/v) (phase B). The separation linear gradient was 0 min (88%, B) −20 min (85%, B) −30 min (80%, B) −40 min (20%, B). The flow rate was: 80 mL/min. We collected secondary fractions based on mass spectrum and UV peaks, also, we collected the fractions between peaks, named as Fn-P1, Fn-P2… Fn-Pn.

### NMR and MS analysis of the active compound

NMR spectra were detected by using NRM spectrometer(Bruker, USA), samples dissolved in D_2_O. MS spectra were obtained by using Orbitrap Elite mass spectrometry under positive mode with 180 voltages (Thermo Fisher Scientic, MA, USA).

### Data visualization

For each fraction’s inhibitory rates were extracted for similarity analysis. For visualization, the inhibitory rates were color coded to illustrate relative differences (red: high inhibitory; black: control). Every row and column carries equal weight. The Ward hierarchical clustering algorithm and Euclidean distance metrics were used for clustering the PN fractions. SSB in the vehicle at a concentration that equals to those for all fractions was also employed as a negative control. Each fraction was assayed at three replicates.

### Inhibition of ACE Activity in Rat Tissues

The procedure was modified base on Meng’s research^44^. Briefly, kidneys from male SD rats were homogenized in 20 mM pH 8.3 potassium phosphate solution with 10 µg/mL leupeptin, 2 µg/mL aprotinin, and 10 µg/mL pepstatin A. All these mixture were centrifuged 10 min at 600 g, washed, and then collected the supernatants, and the supernatants was centrifuged for 20 min at 40 000 g. Pellets were then diluted to in 100 mM potassium phosphate to 0.2 g tissue/mL to obtain the membrane suspension. The membrane suspension was used for measurements. To measure the activity of ACE, 100 µL of membrane suspensions were preincubated with 50 µL 100 µM of catechin and Captopril at 37 °C for 30 min respectively. Then 25 µL of 6.88 mM HLL was added and incubated at 37 °C for 1 h. The reaction was terminated by adding 200 µL 12% phosphoric acid. Ethyl acetate were added to extract the hippuric acid. After centrifugating 2 min at 1000 g, a 700 µL aliquot of the ethyl acetate layer was dried under a N_2_ condition. The pellet was then resuspended in 0.1% (v/v) TFA in H_2_O/acetonitrile (75:25, v/v), and the hippuric acid was determined as above.

### Statistical analysis

Data were analyzed by using SPSS.

## Results and Discussion

### Validation of LC/MS-based Assay

#### Calibration carve, recovery and repeatability of the method

One of the aims of this study was to develop a fast,robust and high sensitivity HPLC/MS-based screening procedure for ACEis in crude pine needle extract by applying HHL as a substrate.By using C18 HC column, we obtained superior product separation from crude reaction-mixture samples using a loading flow of 1 mL/min. Additionally, this method showed high sensitivity and wide linear range. The regression equations of three concentration groups are:

$${\rm{Y}}=95.18\ast {\rm{X}}+1.820,{R}^{2}=1.000$$

$${\rm{Y}}=94.55\ast {\rm{X}}+9.631,{R}^{2}=0.9993$$

$${\rm{Y}}=92.45\ast {\rm{X}}+12.03,{R}^{2}=0.9796$$

This demonstrated that the linear relation between peak area and three concentration groups of Hip were good.

Also, this method showed higher recovery with high repeatability (Table 1). The recovery ± SEM was 102.5 ± 1.3%, 99.1 ± 2.6%, 101.9 ± 1.5%, n = 5.

**Table 1:** Repeatability of the method.

	Peak Area(Hip) (n = 5)	
	Continuous injection	Different time points injection
avg	19976	20019
STEDV	173.93	198.32
RSD	0.87%	0.99%

### Determination of ACE activity

The results of HHL hydrolysis by ACE showed it needed a great amount of enzyme to reach a hydrolysis rate of 50%. We found that a steady state was obtained by a Enzyme/Substrate ratio of 2.5 mU/nM, and we moved on with this ratio for all measurements. For the incubation time, different assays demonstrated the HHL hydrolysis was optimum after 30 min incubation.

### Determination of ACE-inhibitory activity of Captopril

**Figure 3:**
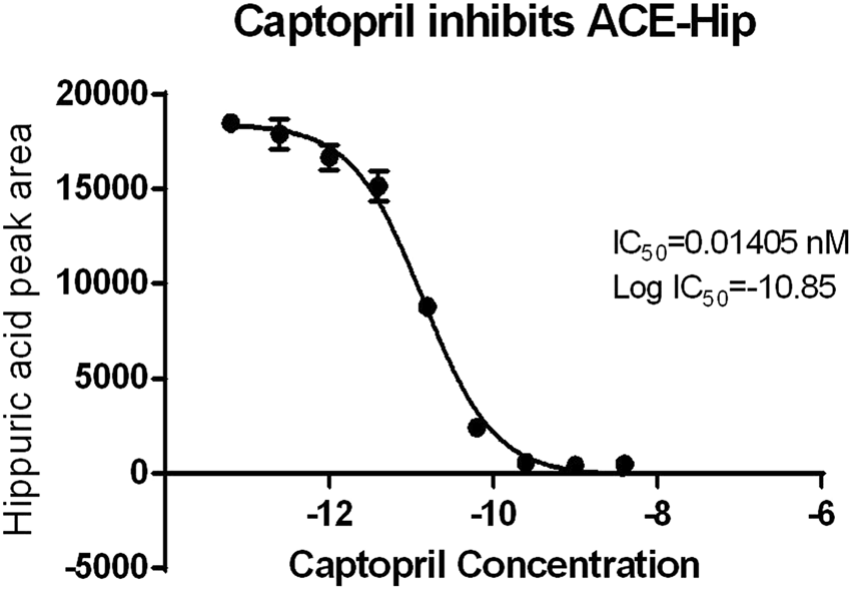
ACE-inhibitory activity of Captopril.

The inhibition of Captopril allowed us to determine a Log IC_50_ value of −10.85 which is corresponding to a concentration of 0.014 nM. The repeated assessments demonstrated high sensibility and high stability of the Captopril in high concentration solution, whereas the stability in low concentration Captopril solution was lower than that of high concentration. This may due to the oxidative degradation at thiol function lead to Captopril- disulphide, which is no ACE inhibiting activity. A low concentration of Captopril is stable only three days at 5 °C^45^. This explained the important standard deviation of the IC_50_ value in low concentration group (Fig. 3).

### The ACE-inhibitory effect profiling of pine needle fractions

**Figure 4:**
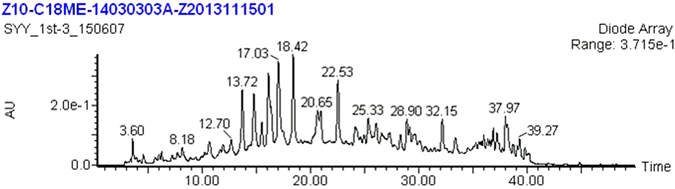
PN first fractionation Analysis.

The extraction and first-dimensional fractionation of pine needle was showed as Fig. 4. We collected each fraction for 2 mins, so there were 20 fractions from each extraction. We repeated the extraction four times, each time 1 g, so total 4 g, and we combined same fractions from 4 times together. We dried and weighted each fraction. We analyzed all twenty fractions as we described in the methods, and found that the components of these fractions were extremely complex.

**Figure 5:**
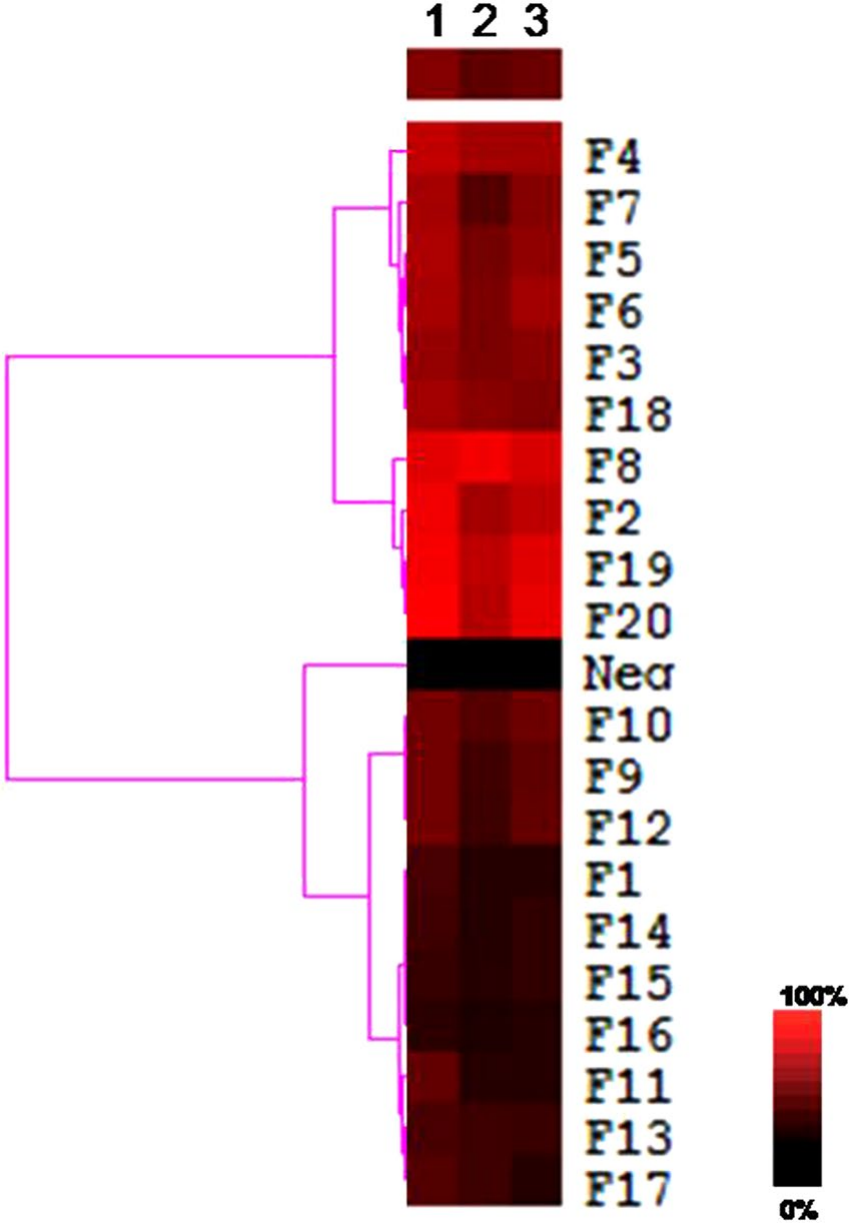
The heat map of PN fractions. This heat map was obtained by using similarity analysis of the inhibition rate of the each fraction. For each inhibition profile, the results were color coded – red: high inhibition effect; black: no inhibition. False color scale bar is included to assist the data visualization. N = 3 (x axis are 3 repeats).

**Figure 6:**
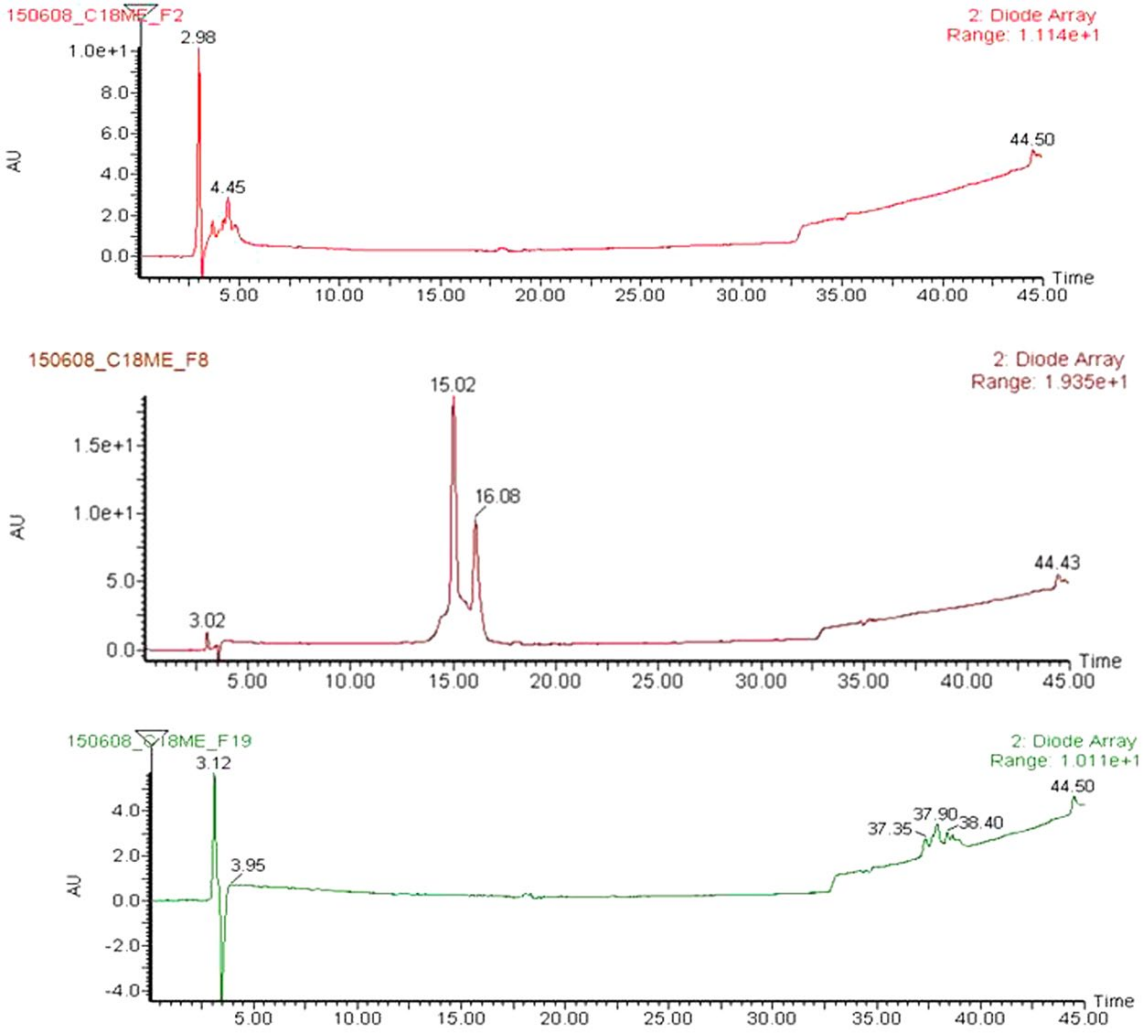
LC-MS analysis of fraction F2, F8, F19 (upper, middle and lower). Mobile phase A is 0.1% (v) TFA in water and B is 0.1% (v) FA in ACN. The gradient of separations was 0 min (5%, B)−15 min (95%, B). The flow rate was 1 ml/min.

The ACE activity assay afforded by LC/MS was used to profile all these first-dimensional fractionations above. Each fraction was analyzed with three replicates at 10 mg/mL. Similarity analysis using Ward hierarchical clustering algorithm and Euclidean distance metrics led to an ACEi-bioactivity phenotypic heat map. This similarity analysis categorized 20 fractions and one negative control into three major clusters (Figs 5 and 6).

Several interesting features emerged. First, among 20 fractions, hit rate was relatively high. There are almost half of the fractions showed inhibitory active (F4, 7, 5, 6, 3, 18, 8, 2, 19, 20). This hit rate was partial because of the complexity of bioactive constituents in PN extract, and also because of the crude fractionation approach used. This procedure may lead to unevenly collection of compound in multiple continuously fractions. Secondly, several of the ten fractions that showed inhibition were found mainly associated two separate fraction groups. But the similarity analysis result showed the consecutive fractions actually were not with same similarity, and the F8 with the highest inhibitory active was independent of neither groups. We speculated that the consecutive fraction group may with different compounds to be responsible for the activity and the group F2, 8, 19, 10 were the higher possibility is for the ACE inhibitory activity of PN (Fig. 5).

### Identification of bioactive compound(s) in F8

We identified the active compounds in F8 using several methods, since F8 displayed the greatest inhibition effect and less difficulty of separation among the active fractions. Firstly, we compared the LC-MS profiles of the 20 fractions (data did not show here) by monitoring UV absorbance and MS (ESI+). Results demonstrated that there were overlaps in UV absorbance and total ion chromatograms among these fractions, though each crude fraction is a complex mixture and some fractions like F2 had weak retain, even some fractions like F19 with extremely low content. Based on the inhibitory effect, the peak around 16–18 min most likely contains the most potent active compound.

Secondly, the values of Hill coefficient obtained for Captopril and the F8 were 1.83 and 1.91 respectively, expressing well the property of the ACE to possess two binding sites for low molecular weight inhibitors. This result confirms the inhibitors present in the bioactive compounds in PN fractions are low-molecular molecules.

**Figure 7:**
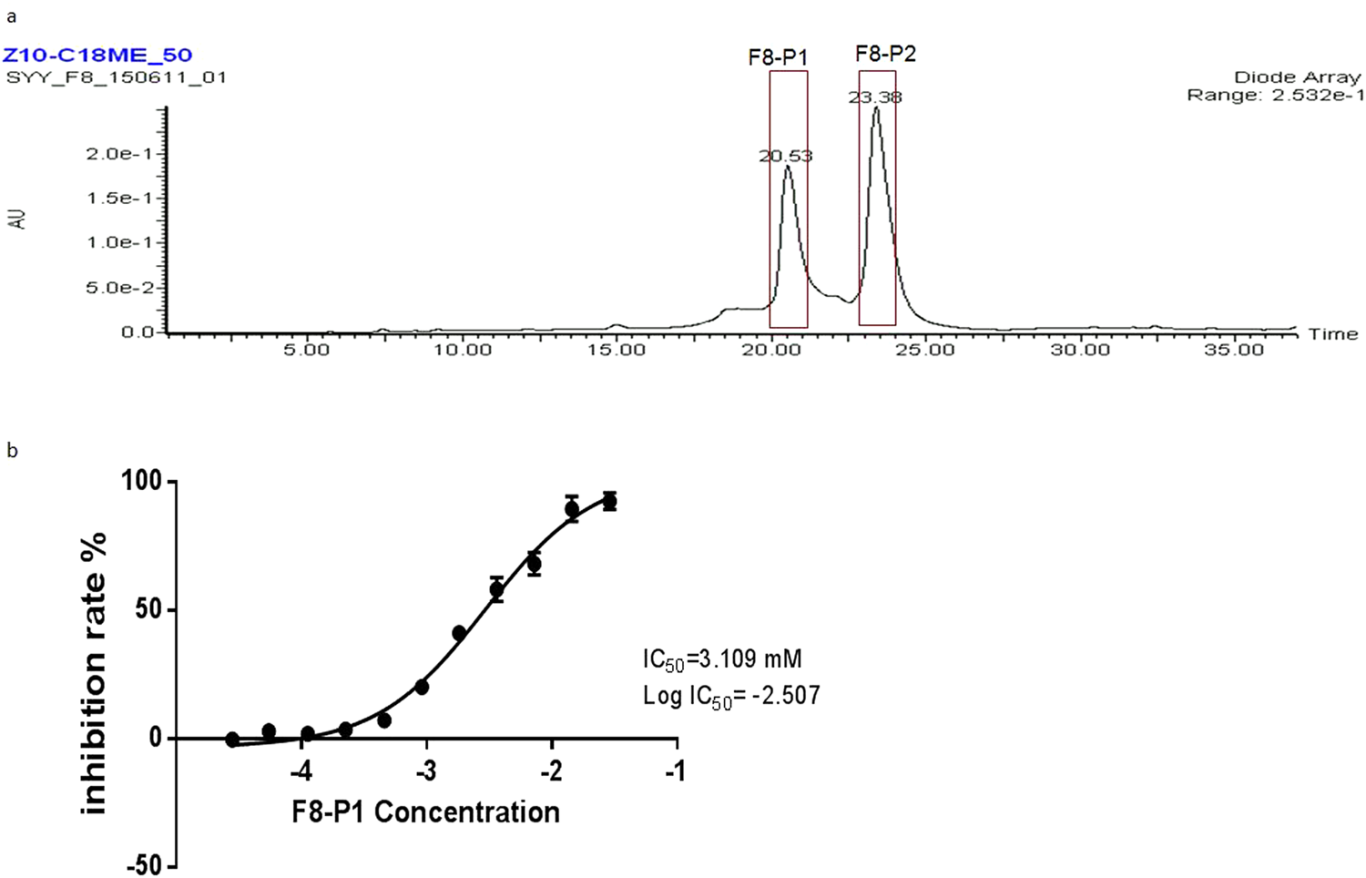
Analysis of the F8. (**a**) The two secondary fractions were collected (in red boxes). (**b**) Dose response of F8. Data represents mean ± SEM. (n = 3).

Third, we used HPLC to prepare secondary fractions from F8 based on both UV and chromatograms. Two secondary fractions were named F8-P1 and F8-P2, corresponding to the peaks at 20.53 and 23.38 min (Fig. 7). We finally tested the dose responses of these two secondary fractions and found that only F8-P1 trigged an inhibitory effect which observed in the initial screening, and led to a dose response with log EC_50_ of 3.1 mM (n = 3) (Fig. 7a and b).

**Figure 8:**
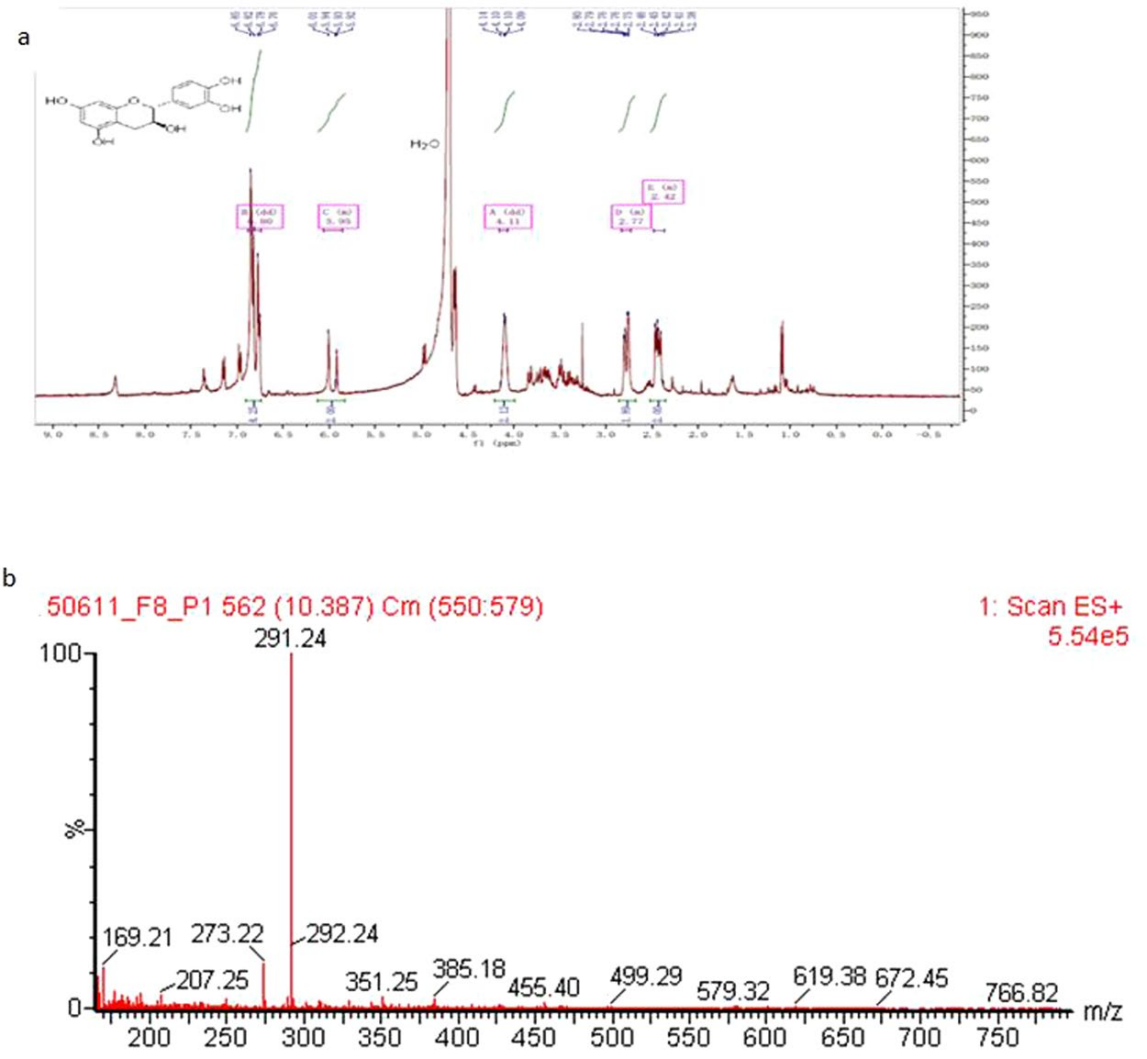
NMR (**a**) and HRMS analysis (**b**) of F8-P1.

Forth, we characterized F8-P1 using HRMS and NMR. HRMS results showed that C2 has an m/z of 291.24 (Fig. 8a). 1H-NMR spectra of F8-P1 further confirmed that it is catechin (1H-NMR in D2O): 2.56–2.81 (d, 2 H), 4.86–4.88 (m, 2 H), 5.02 (s, 1 H), 5.89–5.91 (s, 2 H), 6.65–6.76 (m, 3 H), 9.48 (s, 4 H) (Fig. 8b).

Fifth, since catechin was the bioactive chemical of F8, we selected ion m/z of 291 as the targeted species to examine the distribution of catechin in all fractions. LC/MS analysis demonstrated that the targeted molecule, catechin, in all fractions groups is enriched in F8 with the peak at 15.22 min.

All these together, these results demonstrated that the bioactive compound in pine needle extract being ACE-inhibitory active is catechin.

### Effect of Catechin on ACE Inhibition in Rat Tissues

To access ACE inhibition in a environment close to physiological condition, organ membrane suspension isolated from rat kidney was incubated with catechin and Captopril (positive control). The inhibition of equal volumes of captopril and catechin was 90% and 43.2% respectively. As observed for the purified enzyme, the ACE activity in kidney membrane suspensions was inhibited by 100 µM of catechin (p < 0.001). The inhibition of ACE by catechin was 45% (p < 0.001). The inhibition of ACE was also observed in tissue membrane suspension of rat testes and lungs. Catechin inhibited 56% of ACE activity in testes and 30% of ACE activity in lung respectively.

## Conclusions

In the present study we have presented bioactivity-guided fractionation profiling strategy to identify bioactive compounds of pine needle, and to determine its action mechanism. We figured out there was high amount of catechin in the plant extract. The presence of catechin in pine needle associate to its anti-hypertension curative effect observed in clinical. Our study highlights the potential use of pine needle even other medicinal plants in drug or health food industry, its therapeutic benefits and bioactive compounds behoove further investigations.

## References

[1] Alissa, E. M. & Ferns, G. A. Functional foods and nutraceuticals in the primary prevention of cardiovascular diseases. J. Nutr Metab. 569486 (2012). 10.1155/2012/569486 PMC3335253 22570771

[2] El-Atat F, McFarlane SI, Sowers JR (2004). Diabetes, hypertension, and cardiovascular derangements: pathophysiology and management. Curr Hypertens Rep..

[3] Winter KH, Tuttle LA, Viera AJ (2013). Hypertension. Prim Care..

[4] Faraco G, Iadecola C (2013). Hypertension: a harbinger of stroke and dementia. Hypertension..

[5] Sun ZA (2015). arterial stiffness, and hypertension. Hypertension..

[6] Ezzati, M. *et al*. Rethinking the “diseases of affluence” paradigm: global patterns of nutritional risks in relation to economic development. PLoS Med. e133 (2005). 10.1371/journal.pmed.0020133 PMC1088287 15916467

[7] Griendling KK, Tsuda T, Berk BC, Alexander RW (1989). Angiotensin II stimulation of vascular smooth muscle cells. Secondary signalling mechanisms. Am J Hypertens..

[8] Griendling KK (1988). Secondary signalling mechanisms in angiotensin II-stimulated vascular smooth muscle cells. Clin Exp Pharmacol Physiol..

[9] Atlas SA (2007). The renin-angiotensin aldosterone system: pathophysiological role and pharmacologic inhibition. J. Manag Care Pharm..

[10] Fotherby MD, Panayiotou B (1999). Antihypertensive therapy in the prevention of stroke: what, when and for whom?. Drugs..

[11] Chuah HS, O’Donnell D (2012). Angiotensin-converting enzyme inhibitor and visceral angio-oedema. Emerg Med Australas..

[12] Mukamal KJ, Ghimire S, Pandey R, Fiarman GS, Gautam S (2012). Angiotensin-converting enzyme inhibitors, angiotensin-receptor blockers, and risk of appendicitis. Ann Epidemiol..

[13] Kuhlen JL, Forcucci J (2012). Angiotensin-converting enzyme inhibitor-induced unilateral tongue angioedema. Am J Med Sci..

[14] Thalanayar PM, Ghobrial I, Lubin F, Karnik R, Bhasin R (2014). Drug-induced visceral angioedema. J Community Hosp Intern Med Perspect..

[15] Somanadhan B (1999). An ethnopharmacological survey for potential angiotensin converting enzyme inhibitors from Indian medicinal plants. J. Ethnopharmacol..

[16] Nyman U (1998). Ethnomedical information and *in vitro* screening for angiotensin-converting enzyme inhibition of plants utilized as traditional medicines in Gujarat, Rajasthan and Kerala (India). J. Ethnopharmacol..

[17] Duncan AC, Jager AK, van Staden J (1999). Screening of Zulu medicinal plants for angiotensin converting enzyme (ACE) inhibitors. J. Ethnopharmacol..

[18] Adsersen A, Adsersen H (1997). Plants from Réunion Island with alleged antihypertensive and diuretic effects–an experimental and ethnobotanical evaluation. J. Ethnopharmacol..

[19] He J, Li YL (2015). Ginsenoside Rg1 downregulates the shear stress induced MCP-1 expression by inhibiting MAPK signaling pathway. Am J Chin Med..

[20] He J (2011). Effect of Mesima Reishi UE-1 on invasion of human ovarian cancer cells *in vitro*. Afr. J Biotech..

[21] Corson TW, Crews CM (2007). Molecular understanding and modern application of traditional medicines: triumphs and trials. Cell..

[22] Newman DJ (2008). Natural products as leads to potential drugs: an old process or the new hope for drug discovery?. J. Med. Chem..

[23] Li JW, Vederas JC (2009). Drug discovery and natural products: end of an era or an endless frontier?. Science..

[24] Newman DJ, Cragg GM (2012). Natural products as sources of new drugs over the 30 years from 1981 to 2010. J. Nat. Prod..

[25] Zhu F (2012). Drug discovery prospect from untapped species: indications from approved natural product drugs. PLoS One..

[26] Weber W, Fussenegger M (2009). The impact of synthetic biology on drug discovery. Drug Discovery Today..

[27] Swinney DC, Anthony J (2011). How were new medicines discovered?. Nat. Rev. Drug Discovery..

[28] Huang WY, Davidge ST, Wu J (2013). Bioactive natural constituents from food sources-potential use in hypertension prevention and treatment. Crit Rev Food Sci Nutr..

[29] Cushman, D. W., Cheung, H. S. Spectrophotometric assay and properties of the angiotensin-converting enzyme of rabbit lung. Biochemical Pharmacology. 1637–1648 (1971). 10.1016/0006-2952(71)90292-9 4355305

[30] Doig MT, Smiley JW (1993). Direct injection assay of angiotensin-converting enzyme by high-performance liquid chromatography using a shielded hydrophobic phase column. J. Chromatogr..

[31] Mehanna AS, Dowling M (1999). Liquid chromatographic determination of hippuric acid for the evaluation of ethacrynic acid as angiotensin converting enzyme inhibitor. J. Pharm Biomed Anal..

[32] Van Elswijk (2003). Rapid detection and identification of angiotensin-converting enzyme inhibitors by on-line liquid chromatography-biochemical detection, coupled to electrospray mass spectrometry. J. Chromatogr A..

[33] Liesener A, Karst U (2005). Monitoring enzymatic conversions by mass spectrometry: a critical review. Anal Bioanal Chem..

[34] Elased KM, Cool DR, Morris M (2005). Novel mass spectrometric methods for evaluation of plasma angiotensin converting enzyme 1 and renin activity. Hypertension..

[35] Elased KM, Cunha TS, Gurley SB, Coffman TM, Morris M (2006). New mass spectrometric assay for angiotensin-converting enzyme 2 activity. Hypertension..

[36] Duncan MW, Roder H, Hunsucker SW (2008). Quantitative matrix-assisted laser desorption/ionization mass spectrometry. Brief Funct. Genomic Proteomic..

[37] Pezzuto, J. M. Plant-derived anticancer agents. Biochem Pharmacol. 24; 53: 121–133 (1997). 10.1016/s0006-2952(96)00654-5 9037244

[38] Chen, L. *et al*. Bioactivity-Guided Fractionation of an Antidiarrheal Chinese Herb Rhodiola kirilowii as Inhibitors of Cystic Fibrosis Transmembrane Conductance Regulator. PLoS One. 6; 10: e0119122 (2015). 10.1371/journal.pone.0119122 PMC4352019 25747701

[39] Liu X (2004). Pycnogenol, French maritime pine bark extract, improves endothelial function of hypertensive patients. Life Sci..

[40] Bakhtin IV (2006). Efficiency of Siberian pine oil in complex treating of people ill with benign hypertension. Vopr Pitan..

[41] Kwak CJ (2009). Antihypertensive effect of French maritime pine bark extract (Flavangenol): possible involvement of endothelial nitric oxide-dependent vasorelaxation. J Hypertens..

[42] Schoonees A, Visser J, Musekiwa A, Volmink J (2012). Pycnogenol® (extract of French maritime pine bark) for the treatment of chronic disorders. Cochrane Database Syst Rev..

[43] Wronique l, Karine R, Laure T, Dominique H, Patrick A (2010). A HPLC-UV method for the determination of angiotensin I-converting enzyme (ACE) inhibitory activity. Food Chemistry..

[44] Meng QC, Balcells E, Dell’Italia L, Durand J, Oparil S (1995). Sensitive method for quantitation of angiotensin-converting enzyme (ACE) activity in tissue. Biochem. Pharmacol..

[45] Anaizi. NH, Swenson C (1993). Instability of aqueous Captopril solutions. American Journal of Hospital Pharmacy..

